# An unusual complication following endoscopic ultrasound-directed transgastric ERCP in a patient with a history of bariatric surgery

**DOI:** 10.1055/a-2291-9505

**Published:** 2024-04-09

**Authors:** Daniele Balducci, Clément Archimbaud, Jean-Philippe Ratone, Yanis Dahel, Solene Hoibian, Fabrice Caillol, Marc Giovannini

**Affiliations:** 1 56181 Endoscopy Unit, Institut Paoli-Calmettes, Marseille, France; 2 9294 Clinic of Gastroenterology, Hepatology, and Emergency Digestive Endoscopy, Università Politecnica delle Marche, Ancona, Italy

We present the case of a 56-year-old woman with a history of sleeve gastrectomy and gastric bypass who developed an atypical complication following gastro–gastric anastomosis during an endoscopic ultrasound (EUS)-directed transgastric endoscopic retrograde cholangiopancreatography (ERCP) procedure (EDGE) to access the biliary tract. The patient had undergone sleeve gastrectomy followed by gastric bypass 7 years previously for severe gastroesophageal reflux disease. She was referred to our center for management of symptomatic biliary tract stones. EUS confirmed the presence of biliary lithiasis and EDGE was planned for biliary access.

Having obtained the patient’s consent, we proceeded with EDGE. During the first procedure, the excluded stomach was punctured, and a lumen-apposing metal stent (LAMS; Axios, 15 mm) was successfully placed, without any apparent complications – in particular there was no visible bleeding. Because of the patient’s previous sleeve gastrectomy, the excluded stomach had a smaller volume and a thicker wall than the typical excluded stomach. The procedure required more traction and thrust than the standard procedure, along with the use of a pure CUT current of 10 to cross the gastric wall ( Video 1 ).

Procedural steps performed in the creation of a fistula tract between the gastric pouch and the excluded stomach, along with management of the complication.Video 1

After the procedure, the patient developed abdominal pain and hemorrhagic shock, and immediate intervention was therefore needed. A computed tomography scan revealed a hemoperitoneum with active contrast leakage from a branch of the superior mesenteric artery distant to the LAMS, and emergency embolization was performed ( Video 1 ). A sphincterotomy of the main biliary tract was undertaken 2 weeks later, and the LAMS was subsequently removed. The patient's recovery was uneventful, and she resumed normal activities.

Endoscopy_UCTN_Code_CPL_1AL_2AB

